# A Dynamic Role of Mastermind-Like 1: A Journey Through the Main (Path)ways Between Development and Cancer

**DOI:** 10.3389/fcell.2020.613557

**Published:** 2020-12-21

**Authors:** Sabrina Zema, Maria Pelullo, Francesca Nardozza, Maria Pia Felli, Isabella Screpanti, Diana Bellavia

**Affiliations:** ^1^Department of Medico-Surgical Sciences and Biotechnology, Sapienza University, Latina, Italy; ^2^Center for Life Nano Science@Sapienza, Istituto Italiano di Tecnologia, Rome, Italy; ^3^Department of Molecular Medicine, Sapienza University, Rome, Italy; ^4^Department of Experimental Medicine, Sapienza University, Rome, Italy

**Keywords:** MAML, Notch, Hedgehog, Hippo, signaling pathway, development, coactivator, cancer

## Abstract

Major signaling pathways, such as Notch, Hedgehog (Hh), Wnt/β-catenin and Hippo, are targeted by a plethora of physiological and pathological stimuli, ultimately resulting in the modulation of genes that act coordinately to establish specific biological processes. Many biological programs are strictly controlled by the assembly of multiprotein complexes into the nucleus, where a regulated recruitment of specific transcription factors and coactivators on gene promoter region leads to different transcriptional outcomes. MAML1 results to be a versatile coactivator, able to set up synergistic interlinking with pivotal signaling cascades and able to coordinate the network of cross-talking pathways. Accordingly, despite its original identification as a component of the Notch signaling pathway, several recent reports suggest a more articulated role for MAML1 protein, showing that it is able to sustain/empower Wnt/β-catenin, Hh and Hippo pathways, in a Notch-independent manner. For this reason, MAML1 may be associated to a molecular “switch”, with the function to control the activation of major signaling pathways, triggering in this way critical biological processes during embryonic and post-natal life. In this review, we summarize the current knowledge about the pleiotropic role played by MAML proteins, in particular MAML1, and we recapitulate how it takes part actively in physiological and pathological signaling networks. On this point, we also discuss the contribution of MAML proteins to malignant transformation. Accordingly, genetic alterations or impaired expression of MAML proteins may lead to a deregulated crosstalk among the pathways, culminating in a series of pathological disorders, including cancer development. Given their central role, a better knowledge of the molecular mechanisms that regulate the interplay of MAML proteins with several signaling pathways involved in tumorigenesis may open up novel opportunities for an attractive molecular targeted anticancer therapy.

## Introduction

Developmental signaling pathways, such as Notch, Wnt/β-catenin, Hedgehog (Hh), and Hippo, are highly conserved in multicellular organisms. These signaling cascades play a significant role in embryonic development, to establish body plan and they are also involved in the onset/progression of several cancers. In eukaryotic cells, extracellular and intracellular signals can stimulate these pathways, which convert the stimuli in specific transcriptional events inside the cell. The transcriptional activation of eukaryotic genes is a multistep process, controlled by tightly regulated assembly of multiprotein complexes, constituted by transcription factors and co-regulators proteins (coactivators or co repressors) recruited on specific enhancers and promoters to determine the final outcome (Larivière et al., 2012). The transcription factors contain a specific DNA-binding domain that directly interacts with the DNA in a sequence-specific fashion, a multimerization domain and a transcription activation domain. In addition to the fundamental role played by transcription factors, several coactivators assemble in response to specific cellular signals. They dock to transcription factors and some of them trigger enzymatic activity to modify chromatin and recruit RNA polymerase II. Therefore, even though transcriptional co-regulators are unable to directly bind to the DNA, they are strongly required to control the transcriptional activation (Swygert and Peterson, 2014) and notably the composition of coactivators inside the complex is itself a dynamic response to signal transduction. Therefore, in response to the stimuli received by the cell, the coactivators are able to modulate the transcriptional outcome with dynamic changes of the components inside multiprotein complexes to generate an accurate and efficient regulation of gene expression (Rosenfeld et al., 2006; Krasnov et al., 2016).

In this review, we focus our attention on Mastermind-like (MAML) transcriptional coactivators family, firstly described as integral part of the Notch signaling pathway (Helms et al., 1999; Petcherski and Kimble, 2000b; Wu et al., 2000; Kitagawa et al., 2001), and now reported as pleiotropic interactors across multiple signaling pathways. Here, we recapitulate the ability of MAML proteins, in particular MAML1, in regulating the major developmental signaling cascades, starting from the canonical role played inside the Notch signaling, until the unexpected Notch-independent role carried out in Wnt/β-catenin (Alves-Guerra et al., 2007), Sonic Hh (Quaranta et al., 2017) and Hippo pathways (Kim et al., 2020). We also describe the relevant role for MAML1 in regulating both physiological or pathological settings, sustained by important transcription factors, as MEF2C (Shen et al., 2006), p53 (Zhao et al., 2007), RelA/NF-κB (Jin et al., 2010), EGR1 (Hansson et al., 2012), and Runx2 (Watanabe et al., 2013). Altogether these observations strongly suggest a central role for MAML1 protein in coordinating the interlinking among the main signaling networks, being able to function as a transcriptional “switch” to trigger specific biological processes. Given its pivotal role as interconnection point among several signaling pathways, it is not surprising that MAML1 protein is directly or indirectly involved in several disorders, including cancer. So, herein we also discuss the contribution of MAML proteins to malignant transformation. We report how *MAML* genes may be regulated by microRNAs and how some DNA viruses can modulate the expression of MAML proteins, thus sustaining viral carcinogenesis. Altogether, these events suggest that several controls are required to regulate the expression/activity of MAML proteins, being involved in several physiological or pathological processes.

## Conventional Role for MAML1 in the Notch Signaling Pathway

### A Brief Overview on Notch Signaling

The Notch pathway is an evolutionarily conserved and finely orchestrated signal transduction, responsible for cell fate determination, embryonic patterning and development, in response to specific cues (Weinmaster, 1998; Artavanis-Tsakonas et al., 1999). The intracellular signals propagated by Notch receptors control a multitude of biological events, including proliferation, differentiation, survival and cellular death along different stages of metazoans development. Accordingly, it is largely demonstrated the involvement of the Notch signaling in regulating several processes, as neurogenesis (Lasky and Wu, 2005), myogenesis (Luo et al., 2005), vasculogenesis (Anderson and Gibbons, 2007), skin development (Lowell et al., 2000), and hematopoiesis (Radtke et al., 2005; Campese et al., 2009). Thus, it is not astonishing that alterations in one or more components of Notch signaling may cause multiple developmental (e.g., Alagille syndrome) and adult (e.g., aortic valve disease) disorders or several cancers, such as T-cell leukemia (Cialfi et al., 2013; Tottone et al., 2019), B-cell leukemia (Rosati et al., 2009; De Falco et al., 2018), breast (Diluvio et al., 2018; Giuli et al., 2019), colorectal (Reedijk et al., 2008; Pelullo et al., 2019a), ovarian (Choi et al., 2008; Ceccarelli et al., 2019), skin cancers (Cialfi et al., 2014; Zhang et al., 2016), and glioma (Catanzaro et al., 2017; Bazzoni and Bentivegna, 2019).

The activation of the Notch receptors (Notch in *Drosophila*, LIN-12, and GPL-1 in *Caenorhabditis elegans* and Notch1-4 in mammals) occurs upon binding to specific ligands (Delta and Serrate in flies, LAG-2 and APX-1 in worms, Jagged1-2 and Delta-like1-3-4 in vertebrates), expressed on neighboring cells. This occurrence determines sequential proteolytic cleavage events, sustained by ADAM (A Disintegrin and Metalloproteinase) and presenilin (PS)/γ-secretase complex, which allow the release of the intracellular domain of Notch receptor (ICN), able to move into the nucleus and to interact with CSL (CBF1/RBP-Jκ in mammals, Su(H) in *Drosophila*, and LAG-1 in *C. elegans)* a DNA-binding transcription factor (Artavanis-Tsakonas et al., 1999; Mumm and Kopan, 2000; Petcherski and Kimble, 2000a; Weinmaster, 2000). Following the most acclaimed model, once in the nucleus, ICN displaces the CSL repressors family, represented by CIR, N-CoR/SMRT, SPEN, SKIP, PDCD4, HDAC, SHARP, and KytoT2 and recruits transcriptional coactivators, including PCAF/GCN5, CBP/p300, and Spt6 transcription elongation factor and Mastermind (MAML) (Borggrefe and Oswald, 2009; Bellavia et al., 2018). A transcriptionally active CSL-ICN-MAML ternary complex assembles into the nucleus and it is able to drive the transcription of several target genes, including the *Hairy/Enhancer of Split* genes [*H/E*(spl); Hes genes in mammals], which encode a family of basic helix-loop-helix (bHLH) transcriptional repressors (Kageyama and Ohtsuka, 1999; Davis and Turner, 2001), p21^WAF/Cip1^ (Rangarajan et al., 2001), Cyclin D1 (Ronchini and Capobianco, 2001), Myc (Palomero et al., 2006; Weng et al., 2006), pTα (Bellavia et al., 2007), as well as components of its own signaling cascade, such as the Notch ligand, Jagged1 (Pelullo et al., 2014).

The ability of the Notch signaling pathway to regulate various physiological and/or pathological processes is easily observed in mammals, where the differential spatio-temporal distribution, both of receptors and ligands, underlies the main developmental processes, in a non-redundant manner. In addition, the recruitment of different Notch modifiers, such as Numb, Fringe, E3 Ub ligases, SUMO, and GSK 3β, modulates the Notch cytoplasmic recycling and protein–protein interactions, further regulating Notch signaling and its transcriptional activity (Giebel and Wodarz, 2012; Palermo et al., 2014; Antila et al., 2018). Finally, the capability of the Notch signaling to interact with other important morphogenetic pathways, such as Hh and Wnt, renders the Notch receptors capable to fully coordinate the expression of different target genes, both directly and indirectly (Pelullo et al., 2019b).

### Mastermind: From Structure to Function in the Canonical Notch Signaling

**FIGURE 1:**
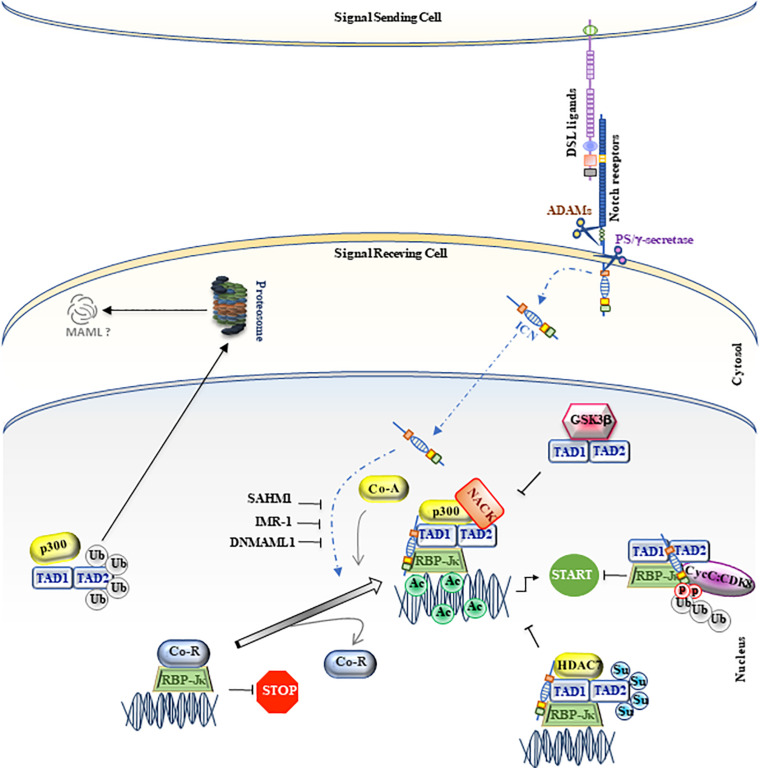
MAML1, as regulator of canonical Notch signaling. A schematic picture of canonical Notch signaling is represented in figure. The MAML1 coactivator is illustrated as TAD1/TAD2. The small colored dots correspond to post-trasductional modifications, such as phosphorylation (light red), ubiquitination (light gray), acetylation (light green) and sumoylation (light blue). The black truncated arrows indicate the negative molecular mechanisms on the pathway. The figure is widely discussed in the text.

*Drosophila mastermind* (DMam) is a neurogenic gene genetically associated with Notch function and isolated in early 1980 (Lehmann et al., 1983; Campos-Ortega et al., 1984; de-la-Concha et al., 1988). Firstly, DMam is detected on polytene chromosomes, able to colocalize with Groucho corepressor protein, suggesting that DMam is a nuclear protein implicated in transcriptional regulation (Smoller et al., 1990; Bettler et al., 1996). After, a Mastermind protein structurally distinct from *Drosophila*, but functionally similar, was identified in *C. elegans* and named Sel8 or LAG-3 (Petcherski and Kimble, 2000a,b). Concurrently, the human homolog of DMam was isolated and denominated Mastermind-like 1 (MAML1) (Wu et al., 2000). Few years later, two other members of Mastermind-like family (MAML2 and MAML3), which share a significant aminoacidic homology with the harbinger MAML1, are identified and characterized. The human *MAML1-2* and *-3* genes are located on chromosome 5q35.3, 11q22.3, and 4q28.3 and code for proteins of 108, 125, and 115 kDa, respectively. Based on biochemical and cellular studies, the MAML proteins are assimilated to transcriptional coactivators, characterized by unusual features typical to regulatory proteins, e.g., polyglutamines, transcriptional activation domains, and clusters of charged amino-acids that bind other proteins (Smoller et al., 1990; Bettler et al., 1996; Lin et al., 2002; Wu et al., 2002). In particular, MAML1 protein is characterized by one basic (ranging from 1 to 228 residues) and two acidic conserved domains (spanning residues 263 to 276 and 990 to 1016), located at the N- and C-terminal regions, which are, respectively, involved in ICN binding and in Notch-mediated transcriptional activation. Furthermore, two transactivation domains (TAD) are present: TAD1, located at the N-terminal region, ranging from 75 to 300 aa, and TAD2 that extends from 303 to 1016 aa at the C-terminal domain. TAD1 is involved in the nuclear localization of MAML1 through its NLS (nuclear localization sequence; ranging from 135 to 141 aa), peculiarly in the nuclear bodies, which probably represent nuclear zones transcriptionally active (Wu et al., 2002; Quaranta et al., 2017). Of note, the TAD1 domain is essential for the formation of the ICN-CSL-MAML1 ternary complex, which binds the promoters of specific target genes and determines their transcriptional activation (Kitagawa et al., 2001; Fryer et al., 2002; Nam et al., 2003). Intriguingly, the tri-dimensional structure of ICN-CSL-MAML ternary complex shows that only when the BTD (β-trefoildomain) domain of CSL and the RAM (RBP-Jκ associated molecule) domain of ICN are bound, an allosteric change occurs and generates a “binding groove,” where the accommodation of MAML1 protein is allowed. Then, MAML1 adopts an elongated α-helical shape that assures to its N-terminal domain the ability to a double touch with the RHR-C domain of CSL and the ankyrin domain of ICN (Kovall and Hendrickson, 2004; Nam et al., 2006). Then, according to the stepwise assembly of the Notch ternary complex, MAML1 is able to recruit other coactivators, such as histone acetyl-transferases (HATs) p300 and PCAF, *vi*a a direct binding through TAD1 domain. These events determine histone H3 and H4 acetylation at lysine residues, permitting the transcriptional activation of Notch target genes (Fryer et al., 2002; Wallberg et al., 2002). In detail, the N-terminal domain of MAML1 is able to interact with p300 and the p300-MAML1 complex allows the histone H3 and H4 acetylation, resulting in the transcription activation. Of note, MAML1 enhances p300 auto-acetylation and this event coincides with the translocation of p300-MAML1/acetylated histones in nuclear bodies. On the other side, MAML1 itself is a substrate of p300 activity at two conserved pair of lysine residues (Lys^138^/Lys^139^ and Lys^188^/Lys^189^). The coactivator p300 interacts with MAML1 through a proline-rich motif (PXPAAPAP) (Saint Just Ribeiro et al., 2007; Hansson et al., 2009), at residues corresponding to 81–87. Notably, the proline rich motif of MAML1 is not sufficient to stimulate p300 histone acetylation activity. In fact, an additional fragment of MAML1 (151 to 350 residues) is required for recruiting p300 acetyltransferase activity (Rogers et al., 2020). Recent is the finding that MAML1, *via* p300, is also able to recruit a new coactivator, NACK, on the ternary complex that, in turns, enrolls the RNA polymerase II to bind to the promoter region of Hes1, the known Notch target gene (Jin et al., 2017). Altogether these observations suggest that the MAML1 TAD1 domain (residues 75 to 301) is required to sustain Notch-dependent transcription (Figure 1).

In addition, the crucial role of Mastermind1 in driving the Notch transcriptional activation is demonstrated by using MAML1 dominant negative mutants (DNMAML1), without the N-terminal domain. These variant fragments are able to inactivate the ICN-CSL-MAML ternary complex, by competing with the wild-type forms, thus allowing Notch-signaling to be switched-off (Nam et al., 2003; McElhinny et al., 2008).

Likewise, glycogen synthase kinase 3 β (GSK3β) directly interacts with MAML1 N-terminus, but it is able to decrease Notch transcriptional activity, by inhibiting MAML1-dependent histone acetylation (Saint Just Ribeiro et al., 2009; Figure 1).

Interesting is the observation that the TAD1 domain mediates also the interaction of MAML1 with components of other pathways, such as MEF2C (Shen et al., 2006), p53 (Zhao et al., 2007), and EGR1 (Hansson et al., 2012), as described below.

**FIGURE 2:**
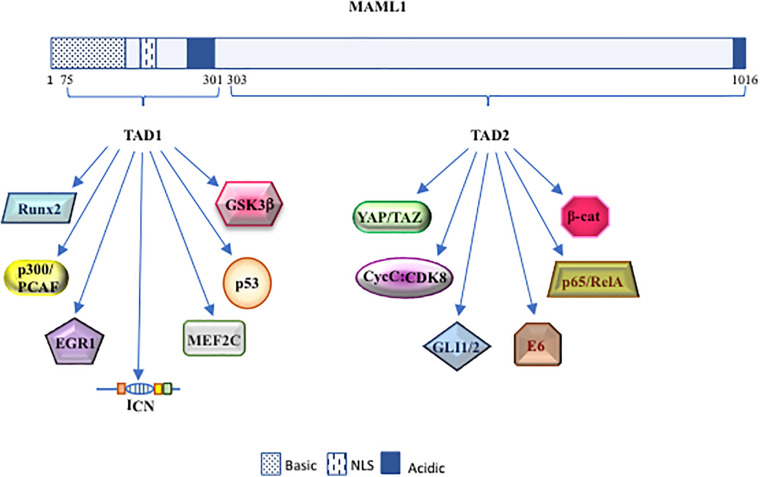
The dynamic role of MAML1. Schematic diagram of MAML1 structure divided in TAD 1 (from 75 to 301 aa) and TAD 2 (from 303 to 1016 aa) domains. The picture highlights the most important interactors of MAML1.

In addition, the MAML1 TAD2 domain, placed at the C-terminal region (spanning 990 to 1,016 residues), contains glutamine-rich sequences that govern the MAML1 transcriptional activity (Fryer et al., 2002). It is not fully clear how the poorly characterized TAD2 domain can regulate Notch expression. It is known that it directly recruits the cyclin C:CDK8 (cyclin-dependent kinase 8) complex that inhibits Notch signaling by promoting phosphorylation-dependent ubiquitination of nuclear ICN and its sequential proteasomal degradation (Fryer et al., 2004). These events require the presence of CBF1/RBP-Jκ, which stabilizes the binding of MAM to ICN (Petcherski and Kimble, 2000b; Fryer et al., 2002; Figures 1, 2).

The mammalian Mastermind proteins are widely expressed in embryonic and adult tissues following a differential spatio/temporal distribution and showing a different affinity/strength to interact with the Notch receptors (Wu et al., 2002). Accordingly, MAML1 deficiency in mice abolishes the development of splenic marginal zone B cells and partially impairs development of early thymocytes (Oyama et al., 2007). Notably, MAML1 null mice does not recapitulate total loss of Notch signaling, and Notch3 null mice show no apparent abnormalities. Conversely, mice null for both MAML1 and MAML3 die during the early organogenetic period, with classic pan-Notch defects, suggesting that the engagement of MAML is essential for Notch signaling (Oyama et al., 2011).

Notably, the differential expression and activity of MAML coactivators may also depend on post-translational modifications, such as SUMOylation and Ubiquitination processes. Lindberg and colleagues demonstrate that the SUMOylation process, at two evolutionary conserved lysine (Lys^217^ and Lys^299^) of Mastermind1, is able to enhance its interaction with HDAC7, which in turn represses the transcriptional activity of MAML1 itself (Lindberg et al., 2010). Similarly, Farshbaf and colleagues identify 8 lysine residues, spanning from 100aa to 800aa of MAML1, which are substrate for p300-mediated ubiquitination in the absence of Notch signaling to maintain low levels of MAML1 in the cell (Farshbaf et al., 2015; Figure 1).

Consistently with the above observations, MAML proteins may have potentially distinct roles in modulating Notch signaling activation in different cell types, taking into account their expression levels and binding force with different Notch receptors, finally contributing to the extraordinary diversity of Notch signaling outcomes in development (Lin et al., 2002; Wu et al., 2002).

Notably, an aberrant Notch signaling is strictly correlated with the onset and/or progression of several types of cancer. A strategy to switch off the oncogenic signaling is to inhibit the assembly of the ICN-CSL-MAML1 ternary complex into the nucleus. To this aim, a synthetic 16-residue peptide from the basic region of MAML1, stapled MAM peptide (SAHM1), is generated (Moellering et al., 2009). SAHM1 is able to associate with ICN and CSL in the Notch transactivation complex and to compete with full-length MAML1, functioning as a dominant negative inhibitor. SAHM1 treatments strongly suppress the expression of Notch target genes, with anti-proliferative effects in leukemic cells and a reduction of tumor progression, both *in vitro* and *in vivo* (Moellering et al., 2009). Moreover, a small molecule inhibitor derived from computer-aided drug design (CADD), named inhibitor of Mastermind Recruitment-1 (IMR-1), is identified. IMR-1 is able to inhibit the recruitment of MAML1 to the Notch-driven transcriptional complex, showing a negative impact on Notch target gene transcription and the ability to abrogate the growth of tumoral cells (Astudillo et al., 2016; Figure 1).

## Unconventional MAML1 Positioning in Major Signaling Pathways at the Crossroad Between Development and Cancer

Firstly identified in the canonical Notch signaling pathway, MAML1 is now recognized as integral component of the main signal transduction cascades, as Wnt/β-Catenin, Sonic Hh and Hippo pathways. In all of these signaling pathways, MAML1 shows a strong ability to convert a plethora of stimuli in several biological processes, such as proliferation, differentiation, tissue development, and tumorigenesis, by modulating the expression of specific target genes.

### Wnt/β-Catenin Signaling Pathway

The Wnt/β-catenin signaling is an evolutionarily conserved pathway, vital for the development of living organisms, being able to modulate important processes, such as differentiation and proliferation (Nusse and Clevers, 2017). The activation of Wnt signaling occurs between adjacent cells that contact each other (Nusse and Clevers, 2017).

In the off-state, β-catenin is localized in the cytosol and prone to proteasomal degradation through β-TrCP activity (He et al., 2004). Wnt proteins are soluble ligands that induce signals through frizzled (Fzd) receptors and LRP5/6, low-density lipoprotein receptor-related co-receptors. Wnt binding induces the recruitment of the Axin to the complex Fzd-LRP5/6 through the scaffold protein Disheveled (Dvd) (Schwarz-Romond et al., 2007; Fiedler et al., 2011; Tauriello et al., 2012), ultimately resulting in β-catenin stabilization (Kishida et al., 1999; Mao et al., 2001; Cliffe et al., 2003), which moves into the nucleus. Once in the nucleus, β-catenin binds to TCF/LEF complex (Behrens et al., 1996), and displaces a repressor complex associated to Groucho, finally activating the transcriptional processes (Cavallo et al., 1998; Nusse and Clevers, 2017).

**FIGURE 3:**
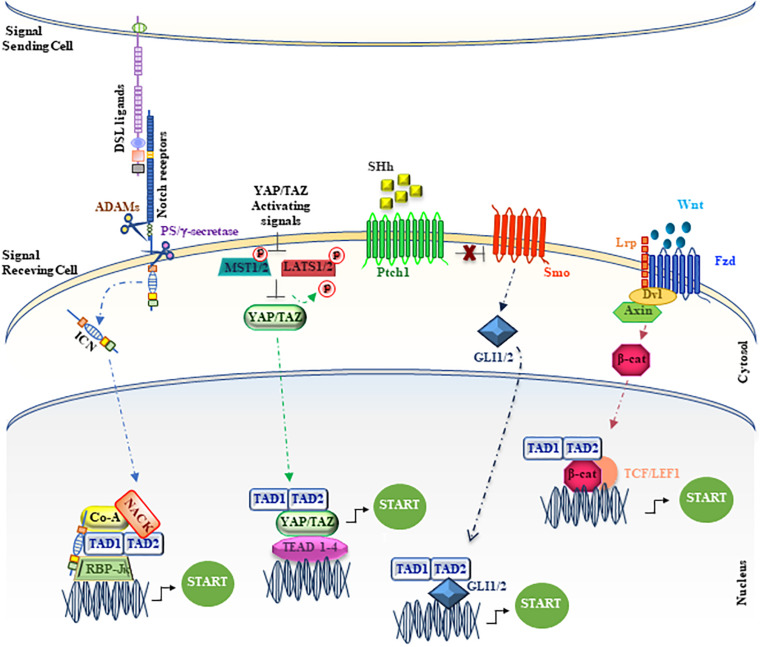
Unconventional positioning of MAML1 in the main signaling pathways. The figure depicts MAML1, indicated as TAD1/TAD2, inside the major signaling cascades, as Notch, YAP/TAZ, SHh and Wnt/β-catenin. The red dots represent phosphate groups. The cartoon is largely discussed in the main text.

Wnt signaling deregulation is linked to the onset of many human cancers, including colon carcinoma and melanoma (Moon et al., 2004; Clevers, 2006), where the oncogenic effects of the Wnt pathway are mediated mainly by β-catenin. Several proteins, including MAML1, can contribute to enhance the transcriptional events, modulating β-catenin/TCF activity (Alves-Guerra et al., 2007). In particular, it is shown that the C-terminal region of MAML1 induces an increase in the transcriptional levels of *cyclin D1* and *c-Myc*, important β-catenin effectors (Alves-Guerra et al., 2007). MAML1 and β-catenin interact each other both *in vitro* and *in vivo*, suggesting that MAML1 is recruited by β-catenin on promoters containing TCF-binding sites to reinforce efficiently the transcriptional activation and to trigger tumor transformation (Figure 3). In fact, MAML1 is required for β-catenin–mediated transcription of specific target genes *in vivo* and is essential for colon carcinoma cell survival. Accordingly, MAML1-depletion induces cell death in colon carcinoma cells and a reduction of Cyclin D1 and c-Myc expression levels, suggesting a pivotal role for MAML1 in colon cancer progression (Alves-Guerra et al., 2007).

### The Sonic Hedgehog Signaling Pathway

Hedgehog Signaling (Hh) is an evolutionary conserved pathway that controls several physiological processes, such as embryonic development, tissue differentiation, cell growth and maintenance of stem cells (Varjosalo and Taipale, 2008; Gorojankina, 2016). Firstly identified in *Drosophila*, *Hh* is a morphogen released during blastoderm stadium, with a specific expression pattern able to sustain the development of anterior-posterior axis in the fly (Nüsslein-Volhard and Wieschaus, 1980; Heemskerk and DiNardo, 1994). In mammals, three paralogs of *Hh* are identified: *Sonic hedgehog (SHh)*, *Indian hedgehog (IHh)*, and *Desert hedgehog (DHh*). The three genes encode for three different proteins with specific functions: SHh plays a key role in the nervous system; IHh takes part in endoderm and bone tissue development and DHh controls the spermatogenesis (Bürglin, 2008).

The canonical Hh pathway is mediated by the interaction between a soluble Hh ligand (SHh, IHh, or DHh) to the Patched (Ptch1) receptor, characterized by twelve transmembrane spanning-domain (Hasanovic and Mus-Veteau, 2018). The ligand/receptor binding determines the co-receptor Smoothened (Smo) release, a class Fzd (class F) G protein-coupled receptor (GPCR), from Ptch1 inhibitory activity (Wang et al., 2013). The Smo activation triggers a powerful signaling cascade, sustained by an intricate network of interacting proteins (Gulino et al., 2012), which ultimately concludes with a dynamic activation and nuclear translocation of downstream GLI1/2 transcription factors to regulate the expression of specific target genes, including GLI1 itself. Interestingly, Hh signaling cascade regulates the transcription both of genes belonging to its own signaling pathway (i.e., *gli1* and *ptch1*) and genes involved in important cellular processes, as proliferation, survival and differentiation (i.e., *cyclinD1* and *D2*, *c-myc*, *hes1*, and *bcl2*) (Hooper and Scott, 2005). In particular, GLI1 protein plays a double role inside the Hh signaling cascade, both as downstream transcriptional effector and as specific target gene of the transduction pathway, representing a feedback loop used to monitor the activation status of the signaling and its effects (Sasaki et al., 1999; Regl et al., 2002). Persistent activation and/or deregulated control mechanisms (Di Marcotullio et al., 2006, 2011; Huntzicker et al., 2006; Zhang et al., 2009; Hui and Angers, 2011; Bufalieri et al., 2019) of Hh signaling pathway are strictly linked to tumorigenesis, maintenance of tumor-initiating/stem cells (Harris et al., 2012), and tumor invasiveness in several types of cancer (Wu et al., 2017). Recent experimental evidence strongly suggests an unconventional role for MAML1, as a novel transcriptional coactivator for Hh/GLI transcription factors, able to enhance the Hh signaling pathway, specifically in stem cells of the *Drosophila* ovary (Vied and Kalderon, 2009) and in cerebellum development in mammals (Quaranta et al., 2017).

The first evidence about the role of DMam as potential factor involved in the regulation of Hh pathway is in follicle stem cells (FSCs) in *Drosophila* ovary (Vied and Kalderon, 2009). More recently, a reliable characterization of MAML1 as partner for GLI proteins has been reported in mammalian cerebellum (Quaranta et al., 2017). Cerebellum development is a finely orchestrated process that begins during the prenatal period with the formation of four principal fissures (Lewis et al., 2004; Sillitoe and Joyner, 2007). The cerebellum granular cell progenitors (GCPs) are responsible for folia formation and for the increase in size of the cerebellum (Lewis et al., 2004; Sudarov and Joyner, 2007). It is known that the cerebellum development is regulated by SHh pathway that sustains GCPs proliferation and cerebellum foliation events (Dahmane and Ruiz-i-Altaba, 1999; Wallace, 1999; Corrales et al., 2004, 2006; Lewis et al., 2004). Impaired SHh signaling results in a reduced GCPs proliferation and the subsequent differentiation, leading to an impaired foliation (Dahmane and Ruiz-i-Altaba, 1999; Lewis et al., 2004). Bio-informatic analysis reported in Differential Atlas database demonstrates that MAML1 expression is more abundant in cerebellum, with respect to other human tissues. Interestingly, MAML1 is necessary to address the nuclear localization of GLI1, in particular into nuclear bodies, and specifically the MAML1 C-terminal region is required to enhance GLI1 transcriptional activity, suggesting a new role for MAML1 TAD2 domain (Figure 3). Accordingly, the SHh signaling pathway results to be strongly hampered in MEFs and GCPs derived from *MAML1*^–/–^ mouse model (Oyama et al., 2007) with a negative impact on GCPs proliferation and cerebellum development *in vivo*, which strictly mimics the features of *SHh*^−⁣/−^ mouse model (Lewis et al., 2004) and suggests a direct involvement of MAML1 as an integral component of SHh signaling in cerebellum development.

Moreover, in primary samples of SHh-driven medulloblastoma, the most frequent childhood brain tumor, MAML1 expression is higher when compared to other medulloblastoma subtypes or healthy cerebellum in *in silico* analysis. Given the above observations, MAML1 behaves as a potent transcriptional coactivator of GLI1 to strongly empower the expression of specific SHh target genes, suggesting that MAML1 may be considered as a novel therapeutic target for developing innovative cancer treatment to contrast cancer growth and proliferation (Quaranta et al., 2017).

### The Hippo Pathway

The Hippo pathway, firstly discovered in *Drosophila*, is extremely conserved in mammals and acts as a key regulator of organ size and tissue homeostasis (Yu et al., 2015). The main effector of the Hippo signaling cascade is YAP/TAZ (Yes-associated protein/transcriptional coactivator with PDZ binding motif) that acts in cooperation with MST1/2 (Mammalian sterile 20-like 1/2 kinases), SAV1 (Salvador1) and LATS1/2 (Large tumor suppressor homolog 1/2 (Pan, 2010; Halder and Johnson, 2011; Yu et al., 2015) core kinase cassette, which shows phosphorylation ability on YAP/TAZ proteins. In particular, YAP and TAZ proteins act as transcriptional co-factors to regulate the expression of target genes. Noteworthy, YAP/TAZ is unable to directly bind onto DNA and the TEAD transcription factors (TEAD1-4), by acting as mediators of YAP/TAZ activity, allow the regulation of YAP/TAZ target genes expression (Zhao et al., 2008). To note, the Hippo pathway, through YAP/TAZ activity, enhances the expression of genes involved in cell proliferation, cell adhesion and cell migration. Different reports highlight the role of Hippo pathway in development, regeneration, and stem cell biology. Alterations in the regulation of the signaling cascade are correlated with the onset and progression of several tumors, such as hepatocellular carcinoma (HCC), pancreatic cancer, colorectal cancer, lung and breast cancer (Lin et al., 2018). Kim and colleagues demonstrate a significant role for MAML proteins to promote YAP/TAZ nuclear localization, showing also the ability to work as YAP/TAZ transcriptional co-factors, sustaining the transcriptional activity (Kim et al., 2020; Figure 3). Both MAML1 and MAML2 enhance nuclear localization and transcriptional activity of YAP/TAZ. Interestingly, MAML1 and MAML2 interaction with YAP/TAZ occurs between the WW domains of YAP/TAZ and an evolutionarily conserved PPxY motif of MAML1 and MAML2 at the C-terminal domain. Notably, MAML1 and MAML2 act as transcriptional coactivators through a trimeric complex with YAP/TAZ and the DNA-binding protein TEAD at the promoters of target genes, such as *ANKRD1* and *CTFG*. A further mechanism of control for MAML1 nuclear localization is given by cell-density. The authors observe a major nuclear abundance of MAML1 and consequently of YAP/TAZ at low cell density (Kim et al., 2020). In particular, miR-30c, induced by high cell density, shows the capability to inversely regulate the expression of MAML1 inside the cell, impairing the nuclear localization and transcriptional activity of YAP/TAZ. Intriguingly, these observations may suggest that the miR-30c-MAML1-YAP/TAZ axis may be a potential therapeutic target for developing novel cancer treatment (Kim et al., 2020).

The role of MAML1 and MAML2, as novel regulators of YAP/TAZ, impinges on the oncogenic properties of the Hippo pathway, involved in the onset and progression of different human cancers (Lin et al., 2018), including colon and lung cancer, where the high levels of MAML1 and MAML2 positively correlate with poor prognosis, emphasizing the MAML1 and MAML2 ability to promote YAP-mediated tumorigenesis. Altogether, the experimental evidence identifies a novel role for MAML1 and MAML2 as transcriptional coactivators of the Hippo pathway.

## MAML1 Interaction With Other Transcription Factors

Beyond the recently identified role played by MAML1 in SHh and YAP/TAZ signaling pathways, it is known that MAML1 can also functionally collaborate with different transcription factors involved in cell differentiation and cancer development, as described below.

### MEF2C

In mammals, MEF2C is a member of *MEF2* (Myocyte enhancer factor 2) gene family, key transcription factors involved in myogenic program during muscle differentiation. MEF2 proteins belong to the MADS box family, characterized by a DNA binding domain, the MADS-box, in the N-terminal region (Black and Olson, 1998). The myogenic program is regulated through MEF2s and MRFs (myogenic regulatory factors) activity. In particular, MEF2C regulates skeletal muscle-specific genes such as *MCK*, *desmin*, and *myogenin* (Black and Olson, 1998). It is reported that MAML1 is able to act as a coactivator for MEF2C transcription factor and to control MEF2C post-translational events to permit the recruitment of other factors, which may contribute to potentiate MEF2C-induced transcription (Shen et al., 2006). The binding site between MAML1 and MEF2C is mapped at the N-terminal domain of MAML1, the same region implicated in the interaction with Notch (Kitagawa et al., 2001; Fryer et al., 2002; Nam et al., 2003). In *MAML1*-null mice, Shen et colleagues observe muscle defects with a decrease of myogenin that results in muscular dystrophy, with a failure of MyoD-induced myogenic differentiation in embryonic fibroblast. The authors suggest a model for MAML1 activity, where in the absence of an activated Notch pathway, MAML1 serves as a transcriptional coactivator for MEF2C to enhance the transcription of genes involved in muscle development, such as muscle creatine kinase (MCK). Upon activation of the Notch signaling, caused by muscle injury, MAML1 switches its role to transcriptional coactivator for Notch, resulting in the expression of the Notch specific target genes, functioning as a molecule with a functional switch property (Shen et al., 2006).

Experimental evidence supports a role for micro RNAs in muscle differentiation (Rao et al., 2006). Actually, the miRNAs contribute to muscle homeostasis regulation and alterations in their expression pattern are found in several muscle disease, such as Duchenne muscular dystrophy and Becker muscular dystrophy (Eisenberg et al., 2007). Additionally, the role of long non-coding RNAs (lncRNA) seems to be very interesting. Notably, it is demonstrated that linc-MD1, a muscle-specific cytoplasmatic lncRNA, is able to regulate MEF2C and MAML1 expression during myoblast differentiation. In particular, linc-MD1 presents miRNA recognition motifs for miR-133 and miR-135 that target MAML1 and MEF2C, respectively (Cesana et al., 2011). In physiological condition, linc-MD1 acts as a decoy for miR-133 and miR-135, providing a positive modulation for MAML1 and MEF2C. By contrast, a depletion of linc-MD1 results in the binding of miR-133 and miR-135 to MAML1 and MEF2C with a consequent down-modulation of their expression levels. On the other side, the overexpression of linc-MD1 induces an up-regulation of MCK, a target of MEF2C and the rescue of linc-MD1 in Duchenne muscle cell results in an increase of MAML1 and MEF2C activity and a partial recovery of the myogenic differentiation program (Cesana et al., 2011). The results suggest a novel regulatory network for MAML1 and its interactors in muscle development. A better knowledge of the mechanisms involved in the MAML1 regulation may improve the therapeutic approach in muscular dystrophies.

### p53

*p53* is a tumor suppressor involved in cell responses to genotoxic stresses through the modulation of cell cycle, apoptosis and senescence expression genes (Lakin and Jackson, 1999; Vousden and Lu, 2002; Dimri, 2005). Upon DNA damage or stress stimuli, the p53 protein expression levels and its activity are regulated by post-translational modifications, including ubiquitination, acetylation, phosphorylation, SUMOylation and methylation (Brooks and Gu, 2003; Bode and Dong, 2004). An additional level of regulation is mediated by the activity of coactivators with histone acetyltransferase activity, such as p300, CBP, PCAF, or others as ADA3, able to regulate p53-mediated transcriptional responses (Sakaguchi et al., 1998; Grossman, 2001; Wang et al., 2001; Kumar et al., 2002; Mazzà et al., 2013). Among the different interactors, MAML1 is recognized as a transcriptional coactivator of p53 and the MAML1/p53 interaction is identified both *in vivo* and *in vitro* (Zhao et al., 2007). Specifically, the N-terminal region of MAML1 (1–302 aa) binds to the DNA binding domain (102–292 aa) of p53 and the ectopic expression of MAML1 enhances the p53-mediated transactivation of specific target genes, as *Bax*, *Gadd45* and *p21*, in a dose-dependent manner. Notably, MAML1 plays a double role on p53, both as a transcriptional coactivator and as a protein modifier to stabilize the half-life of p53 protein upon DNA damage, by promoting phosphorylation and acetylation events (Zhao et al., 2007). Recent reports suggest a crosstalk between MAML1 and p53 in breast cancer development. Interestingly, it is suggested a model where p53 associates with the Notch-driven transcriptional complex, in a MAML1-dependent fashion, to inhibit the Notch-dependent transcription (Yun et al., 2015). Moreover, Shariat Razavi and colleagues demonstrate that the ectopic expression of MAML1 affects the EMT (epithelial mesenchymal transistion) markers expression, which results in an increased E-cadherin expression. This effect decreases the rate of migration in breast cancer cell lines, assuming a cooperation of MAML1 with other pathways, in particular p53, which is able to down-regulate the EMT markers expression (Shariat Razavi et al., 2019). The molecular mechanisms involved in this process have to be further clarified, but these data highlight an interesting role for MAML1 in allowing different outcomes, based on the crosstalk among different pathways and on the cellular context considered.

### EGR1

The transcription factor Early Growth Response-1 (EGR1) is expressed in response to various extracellular signals and diverse stress stimuli. Stimulation by growth factors rapidly induces expression of EGR1, which subsequently leads to the activation of downstream growth pathways (Gitenay and Baron, 2009). EGR1 activity may vary in response to different cellular stimuli: EGR1 can induce apoptosis by stimulating either p53 or PTEN or can promote survival by counteracting p53-dependent apoptosis (Yu et al., 2006). In breast, brain and lung cancers EGR1 acts as a tumor suppressor (Liu et al., 1998). In contrast, in prostate and kidney cancers, EGR1 promotes tumor growth (Yu et al., 2004). The coactivator MAML1 is involved in the regulation of EGR1 mRNA and protein expression (Hansson et al., 2012). MAML1 physically and functionally interacts with EGR1 and together they colocalize in the nuclear bodies. The N-terminal domain of MAML1 (75–127 aa) is important in mediating the synergistic effect with EGR1. EGR1 is a substrate for acetylation process mediated by p300 (Yu et al., 2004), and MAML1 is able to regulate p300 activity by increasing p300 autoacetylation (Hansson et al., 2009). The acetylation process determines increased levels of EGR1 protein, which can strongly stimulate the expression of genes involved in cell growth and survival, playing a crucial role in prostate and kidney cancers (Yu et al., 2004; Gitenay and Baron, 2009). Furthermore, MAML1 may be involved in regulating the stability of EGR1, possibly by increasing the acetylation of EGR1 *via* p300. Finally, public data set reveal a positive correlation among an altered expression of MAML1, EGR1, and p300 in renal clear cell carcinoma (Hansson et al., 2012).

### RelA/NF-κB

The mammalian nuclear factor-κB (NF-κB) family consists of five transcription factors, p50, p52, p65 (RelA), c-Rel, and RelB, which couple with each other to form hetero- and/or homo-dimers (Hayden and Ghosh, 2008). NF-κB regulates different cellular responses, such as proliferation, differentiation, programmed cell death, tumorigenesis, and plays a major role in inflammation and immunity (Chen and Greene, 2004). In the canonical pathway, NF-κB activity is inhibited by IκB regulatory proteins. Different cell stimuli (e.g., pro-inflammatory cytokines, growth factors, or antigen receptor triggering) activate IκB kinases (IKK) that phosphorylate IκB. The inhibitor complex is targeted to the 26S proteasome, resulting in the release of NF-κB that can enter the nucleus and activates the transcription of target genes (Chen and Greene, 2004). Interestingly, MAML1 regulates directly the NF-κB signaling (Jin et al., 2010) by interacting with p65/RelA and enhancing the NF-κB transcriptional activity. On the other side, MAML1 can induce IκBα phosphorylation, with its subsequent ubiquitination and degradation, affecting in this way the stability of the NF-κB inhibitor. These processes lead to an increase in NF-κB transcriptional activity *via* a RelA/MAML1 functional complex. Indeed, when co-expressed with MAML1, RelA switches its subcellular localization, from the cytoplasm to the nuclear bodies. The TAD2 domain, located at the C-terminus of MAML1 is involved in the binding with NF-κB (Jin et al., 2010). Notably, *MAML1*-deficient mice present a high degree of cell death in the liver that correlates with an increase in apoptotic cells, suggesting a defective NF-κB pathway in response to TNFα. Indeed, a normal NF-κB activity is required to protect cells from TNFα-induced cytotoxicity (Beg et al., 1995; Li et al., 1999). These data demonstrate that MAML1 is a p65/NF-κB interactor, able to regulate cell survival. Of note, a crosstalk between MAML1 and NF-κB is also required to control the cell viability in cervical cancer (Kuncharin et al., 2011).

### RUNX2

Runt-related transcription factor 2 (Runx2) is involved in osteoblast differentiation and chondrocyte maturation during bone development. Runx2 belongs to the Runx transcription factor family and is characterized by a DNA-binding runt domain involved in the transcription of target genes (Komori, 2018). Differentiation from mesenchymal cells to osteoblasts is a finely regulated process that is coordinated through Runx2, Sp7, and Wnt signaling. In particular, Sp7 and Wnt control Runx2 enhancer activity to promote expansion of osteoblast progenitors (Kawane et al., 2014). Conversely, the Notch signaling inhibits Runx2-mediated transcription through Hes and Hey proteins, which act as Runx2 repressors (Hilton et al., 2008). A screening system for Runx2 transcriptional co-factors identifies MAML family members as enhancers of Runx2 activity (Watanabe et al., 2013). In particular, residues 343-711aa of MAML1 are essential for Runx2-mediated transcription of osteoblastic differentiation markers *in vitro*. The analysis of *MAML1^–/–^* mice long bones reveals a smaller mineralized region, when compared to the wild type mice. Moreover, histological sections of the area of primary spongiosa of the femoral diaphysis suggest skeletal defects in *MAML1*-null mice, due to an impairment of chondrocyte maturation. Interestingly, the inhibition of the Notch pathway do not affect MAML1-dependent Runx2 transcription, suggesting a Notch-independent role for MAML1 in Runx2 activity in bone development (Watanabe et al., 2013).

## Genetic Alterations of MAML Family Member in Carcinogenesis

Starting from the observation that MAML transcriptional coactivators play a critical role in activating Notch canonical signaling and in sustaining the crosstalk among the major signaling pathways, it is not surprising that MAML proteins deregulation is associated with a number of cancers. Notably, the most common genetic alteration in mucoepidermoid carcinoma (MEC) of the salivary and bronchial glands is a recurrent *t*(11;19) (q21;p13) chromosomal translocation, resulting in a fusion transcript containing the exon 1 of the N-terminus of the MEC translocated 1 gene (MECT1) with the transactivation domain of MAML2 (corresponding to exons two through five) (Nordkvist et al., 1994; Tonon et al., 2003; Bell and El-Naggar, 2013). The MECT1-MAML2 chimeric transcript product is able to activate Notch specific target genes in the absence of any Notch specific ligand (Kaye, 2006; Bell and El-Naggar, 2013), leading to the disruption of normal cell cycle, of differentiation processes and promoting the tumorigenesis (O’Neill, 2009). *MECT1-MAML2* fusion is also found in other types of glandular benign tumors, as Warthin’s tumors (Enlund et al., 2004) and in clear cell hidradenoma of the skin (Behboudi et al., 2005). Likewise, *MLL* is a fusion partner of *MAML2*, resulting from inv(11) (q21q23), in secondary acute myeloid leukemia (AML) and in myelodysplastic syndrome (MDS). In the chimeric protein, the exon seven of *MLL* is fused to exon two of *MAML2*. The MLL-MAML2 fusion protein contributes to carcinogenesis in AML and MDS, by disrupting the Notch signaling pathway (Nemoto et al., 2007). Interestingly, a recurrent *YAP1-MAML2* chimeric transcript is the result of the fusion between TEAD-binding domain of YAP1 region with the transcriptional activation domain of MAML2. YAP1-MAML2 fusion proteins play a role as activators of the Hippo pathway in several cancers, including glioblastoma, ovarian carcinoma, head and neck carcinoma, nasopharyngeal carcinoma and skin cancer (Valouev et al., 2014; Picco et al., 2019). Interestingly, *YAP1-MAML2* chimeric transcript works as oncogenic driver gene, able to trigger the onset/progression of the tumors (Picco et al., 2019). YAP1-MAML2 fused transcript is also identified in poromas and porocarcinoma, where the chimeric protein promotes anchorage-independent growth in epithelial cells (Sekine et al., 2019). Interestingly, high levels of expression of MAML2 are detectable in B cell-derived lymphoma types, when compared to normal tonsillar B cells. An aberrant expression of MAML2 provides an alternative mechanism able to force Notch signaling activation in human lymphoma cells, finally promoting carcinogenesis (Köchert et al., 2011).

MAML3 is also directly involved in the process of carcinogenesis. Notably, MAML3 plays an important role in neuroblastoma progression, where it mediates the resistance to retinoic acid (RA), a drug largely used in neuroblastoma treatment, promoting hyperproliferation of tumoral cells (Heynen et al., 2016). A novel PAX3-MAML3 chimeric protein is found in Biphenotypic sinonasal sarcoma (SNS), a tumor of the nasal and paranasal areas. The translocation *t*(2;4) (q35;q31.1) leads to fusion transcript that associates exon 1–7 of *PAX3* with exon 2–5 of *MAML3*. The chimeric protein works as a potent transactivator of PAX3 response elements with a forced expression of target genes involved in neuroectodermal and myogenic differentiation (Wang et al., 2014).

In addition, an aberrant expression of MAML1 is described in esophageal squamous cell carcinoma (ESCC), human HCC and in head and neck squamous cell carcinoma, where it correlates with the clinicopathological features of tumors, predicting poor prognosis (Forghanifard et al., 2012; Wang et al., 2016; Hashemi Bidokhti et al., 2017; Ardalan Khales et al., 2018; Moghbeli et al., 2019). Recently, novel reports identify a role for MAML1 in T-cell acute lymphoblastic leukemia (T-ALL) in the axis MAML1-SP1-TRIM59, and in breast cancer through the miR-133a-3p/MAML1/DNMT3A positive feedback loop, indicating MAML1 as a potential therapeutic target for these pathological contexts (Cheng et al., 2019; Shi et al., 2019).

Finally, it is noteworthy that *in silico* analysis reveals the presence of some MAML1 mutations in several cancer cell lines. These aberrations are mainly represented by nonsense and frameshift mutations and they could affect both the stability and the folding of MAML1 with functional consequences for Notch signaling (Farshbaf et al., 2015).

## Role of Maml Family Members in Viral Carcinogenesis

Mastermind-like 1 is an interactor of E6 oncoproteins, encoded by bovine papillomavirus type 1 (BPV-1) and by β-human papillomavirus (β-HPV), through the binding mediated by the acid LXXLL α-helical motif, located at the C-terminal acid domain (Brimer et al., 2012; Tan et al., 2012). E6 proteins preferentially associate with MAML1, only BPV1 E6 interacts with MAML3 in addition to MAML1, and CPV7 E6 prefers to bind to MAML2, with respect to MAML1 (Brimer et al., 2017). The E6/MAML interaction determines a viral antagonism on Notch pathway, repressing the transcriptional activation of Notch target genes in epithelial cells. Interestingly, BPV-1 and β-HPV can sequester MAML1 through E6/MAML1 interaction, able to inhibit epithelial differentiation and to promote cancer progression in epithelial cells (Brimer et al., 2012; Tan et al., 2012; South et al., 2014). Accordingly, Notch pathway inactivation is reported in squamous cell carcinoma of the head and neck (Stransky et al., 2011; Wang et al., 2011), suggesting a crucial role of Notch signaling as a tumor suppressor in squamous epithelial cells. In addition, β-HPV5 and β-HPV8 are also associated with lesions and skin cancers in patients suffering of epidermodysplasia verruciformis or in immunosuppressed patients following organ transplantation (Orth et al., 1978). In addition, in neuroblastoma cells, MAML1 cooperates with ORF2, a protein encoded by latency-related (LR)-RNA by bovine herpesvirus 1 (BoHV-1), to stabilize β-catenin and enhance the transcription of target genes. ORF2 and MAML1 co-expression induces cell survival of latently infected neurons with BoHV-1. The stabilization of β-catenin through ORF2 and MAML1 promotes neuronal survival and differentiation, suggesting that MAML1/Wnt signaling relationship is important to maintain BoHV-1 latency and its oncogenic potential (Liu et al., 2016).

## Conclusion

Notch, Wnt/β-catenin, Hh and Hippo are essential transduction pathways that play pleiotropic roles during vertebrate development and their deregulation may sustain the process of tumorigenesis. Each of these major pathways responds to distinct external/internal cellular stimuli that result in a coordinated set of molecular events, whose endpoint is a tight regulation of gene transcription. The broadly accepted paradigm of transcription regulation in eukaryotic cell is a multistep process, strictly controlled by a concerted action of transcription factors and coactivators that build protein assemblies, following a precise sequence for mediating gene activation.

Despite the original identification of MAML1 as a component of Notch signaling pathway, MAML1 may be considered as “master regulator,” showing the ability to integrate different signaling pathways and to activate distinct biological programs in different tissues. Accordingly, MAML1 may activate several other co-regulators inside the canonical Notch signaling, such as p300 and CycC:CDK8, to amplify the signaling process, or interact with components of other signaling pathways, as GLI1/2, β-catenin, and YAP/TAZ (Figure 2). Indeed, the MAML1 coactivator shows an outstanding ability to transcriptionally regulate factors belonging to major signaling pathways, governing important developmental processes. Altogether these observations strongly suggest a central role for MAML1 in coordinating the crosstalk among important signaling pathways, functioning as a master dynamic transcriptional “switch” to sustain biological processes (Figure 3). The crosstalk may result in the recruitment of different coactivators or corepressors following mechanisms of competition or synergy with factors belonging to different signaling pathways and supporting the fine-tuned transcriptional responses, by sustaining the organization of an active multiprotein transcriptional complex. How MAML1 can integrate the external cellular stimuli and address them toward a specific outcome, it is not fully known. Unlike, it is well known that MAML proteins dysregulation is strongly associated with several kinds of human cancers. In this regard, it is intriguing the observation that an aberrant MAML1 expression/activation may disrupt not only the directly related pathway, but also the interconnected signaling cascades, leading to an unbalanced crosstalk that culminates in a series of disorders or cancer development. Certainly, additional investigations are required to better characterize functions of MAML proteins in the different human malignancies where they are involved. Important questions remain to be addressed regarding the molecular characteristics/mechanisms that sustain the MAML1 versatility in coordinating different signaling pathways. This accurate characterization will open numerous possibilities toward new therapeutic approaches by using MAML members as targets in the treatment of cancer.

## Author Contributions

SZ and MP conduced the systematic literature search, wrote the manuscript, and prepared the figures. FN contributed to the literature search and prepared the manuscript. IS and MF reviewed draft of the manuscript. DB put forward the idea of the manuscript and helped with editing. All authors contributed to the article and approved the submitted version.

## Conflict of Interest

The authors declare that the research was conducted in the absence of any commercial or financial relationships that could be construed as a potential conflict of interest.
